# Valedictory Address

**Published:** 1849

**Authors:** Daniel Brainard

**Affiliations:** President of the College


### ARTICLE VII.

*Address to the Graduating Class of Rush Medical College, Session* 1848-9. By Daniel Brainard M.D.; President of the College.

Gentlemen:

In performing the duty which devolves upon me on this occasion, of giving you, along with our final farewell as teachers, some words of advice and encouragement, my thoughts naturally turn again to that profession whose duties you are about to assume. That they are important and difficult beyond the power of most men to perform, none will deny; that they are useful, humane, and charitable beyond those of almost any other profession is not less certain. Our Profession has, however, its deriders; and these have more especially directed their shafts against its younger members; the characters and skill of those more advanced being too be well known to admit of question. It will not, therefore, inappropriate at this time to glance briefly at the claims which medical science and physicians have to public confidence and respect, for it were well that these should be fully impressed upon the minds of the younger members at the commencement of their career.

What has it done for the good of humanity? This is the first question which should be asked respecting any art or science; for watever it may have done to enrich or ennoble its professors, however attractive and delightful it may be in its study or pursuit, it can scarcely be said to possess claims to confidence and respect, still less to the gratitude of man, unless it has conferred essential benefits on the human race.

We inquire, then, whether physicians constitute an essential part of society, or can their services be dispensed with?

.It will not, we suppose, be denied that of all the evils, which afflict man, disease and pain are the first and the greatest. Whether it comes in the private circle, and lays its hand upon the new-born child, blasting the budding hopes of families, or seizes the mature in years and mind, the guide and leader of society; whether it steals in solitary dwellings, a single victim, or stalks abroad as the pestilence, and falls upon terrified cities or nations, disease is still the greatest dread of man. It were beyond the power of the pen to describe the sufferings of a single individual in its grasp, and when taken in the aggregate the imagination of man could but faintly conceive the ills it produces.

> Dire was the tossing, deep the groans,
> Despair tended the sick, and over them
> Death his dart shook but delayed to strike,

There is a class of persons who deny, to a great extent, the necessity of our art, because they say diseases result from a violation of the natural laws, and might be obviated by their observance.

Without, by any means, denying that a violation of the laws of life is a cause of disease, we think that it by no means follows that they can be so observed as to entirely prevent its occurrence. It seems the natural inheritance of man.

When he goes forth to brave the rude climate of the north, his blood is chilled by the freezing blast; in the tropics it is inflamed by the burning sun or poisoned by noxious exhalations. The pursuit of those arts necessary to his existence, leads to disease, and in the accomplishment of those efforts of mind required in all great undertakings, his energies are broken. A world, where the observance of natural laws would secure us against disease, must be one when the body never suffers from heat, cold, accident, or imperfect nutrition; where the mind is never subjected to anxiety or over exertion, or the hopes to disappointment. It would be a Paradise. If you live according to the best known rules, you are not secure against disease; and to reproach men thus placed with a violation of natural laws, is often cruel and unjust. It is not true that nature is the best physician. Inexorable nature, like fate, heeds not the cry of the feeble but rolls on in her iron car, regardless of the victims crushed in her path. It is to art the suffering spirit turns, and not in vain, for relief.

Since, then, disease is a part of the inheritance and history of the human race, it is necessary, that a class of men should exist for its relief—men who should watch over it from the beginning to the end of life, who should search out the causes and nature of man's maladies, and soothe his sufferings in every "lane of life." Such is the required assistance, and it must be admitted that if any class does afford this, it deserves a high rank among the useful classes of society.

It will not be doubted by those conversant with the subject, that the accidents and diseases attendant upon childbirth, are, in a great degree, within the control of art. In no civilized country is the aid of medicine, under such circumstances rejected; and when we take into consideration the debt which society owes for aid so essential to its welfare as that received from the art of accouchment, we see at once its great and inestimable value. Those even, who deny the claims of medical science to respect, fail not to purloin and apply its principles in this instance to as great an extent as their skill will permit.

During the period of chidhood, the watchful care of art is required to guard against the many dangers incident to that tender age; and there is, perhaps, none, even among those who oppose us, who may not be indebted to it for their own health and even their lives.

When we come to consider the different diseases of more advanced years, we find the power of medicine no less conspicuous.

If we take, for example, the class of inflammatory diseases, by which a large part of mankind have always perished, we find irrefragible proofs of the utility and efficiency of medical treatment. Statistics of cases of this kind have been given, and although forgery and falsehood have been resorted to, no reliable account of cases treated by any but the long established and approved methods has been given to show results equally favorable. On the contrary, the constant experience of any practical man, shows the fatal results of delay and inefficient treatment in such cases.

Next in point of fatality to inflammatory diseases are fevers, especially in the western states; the universal experience of those who hear me will bear testimony to their curability under skilful treatment. It is probable that there is no country where the public is so much indebted to the profession for aid in restoring to health the great body of its citizens, as with us, and if the results are still fatal in many cases, it is generally owing to the fact that circumstances do not admit the general application of medical treatment.

We might go through with the whole catalogue of diseases and demonstrate that in each, art has been found either to cure or greatly to mitigate their danger.

But it is not necessary. I cannot, however, forbear adverting to the surgical division of medical science.

More difficult, perhaps, than any other, because coming in contact with incurable affections, it is, in many respects, so admirable and certain as fairly to challenge the admiration of every reasonable mind. What more admirable than the operation of tying arteries on the surface of wounds? what more beautiful than the results of skill applied to the reduction of dislocation or the treatment of fractures? How many thousands has it restored to the sight when blind? to the use of their members when crippled? It is the most humane, beautiful, and admirable of arts, and those who most streuuously deny the utility of medicine, are compelled to admit the value of surgery.

In prevention of diseases, the application of medical science has been at least as efficacious as in their cure. What language can describe, what imagination can conceive, the blessings secured, the ills avoided by the discovery and application of vaccination. We are less sensible of evils prevented than of those from which we are relieved, but when we learn that the rate of mortality at the present time, during the prevalence of the most fearful epidemics, as the cholera, is no greater than the average mortality of ordinary times but little more than half a century since, and find that the average duration of human life has doubled in a century, we shall be prepared to admit the benefit which science applied to health has conferred upon the human race in the prevention of disease.

Among all its benefits there is none more striking than the cure of the disease called mental. An individual restored from insanity to the use of his reason, is a noble trophy presented by science to humanity, and there are annually thousands of this class to whom we might point as evidences of the value of our art. But when we consider that these affections are, in a great degree, transmitted by hereditary descent, and that in proportion as they are neglected they become more numerous and inveterate, and constitute a larger proportion to the whole number, we perceive more fully the value of such treatment. Extinguish the lights which science has shed upon the darkened chambers of the human intellect, and you consign the unfortunate subjects of disease to their former rank as outcasts or criminals—objects of dread and disgust, without sympathy or hope.

It seems strange, if but a small part of these benefits are real, that any should doubt the utility of medical science, or question the claims of physicians to respect. It becomes us, therefore, to examine the objections which may be brought against them.

The first of these, urged from the earliest times, is that medicine is uncertain.

This objection, more or less valid in the earlier stages of the art, has gradually become less so, until at the present time it has no real value whatever. For we have already seen that in regard to the great majority of diseases, the effects of particular modes of treatment are capable of accurate demonstration. If it is in many cases uncertain, the difficulty is in the varying and changeful nature of the objects with which it deals, and not in the principles of the science itself. These, so far as relates to the symptoms of disease and the effects of remedies in given cases, have, within the last half centnry, been brought to an admirable degree of perfection.

But another objection is, that it cannot cure all diseases. Consumption, cancer, typhus fever, and other diseases, are beyond your reach.

To this we reply, that it is not the object or probable end of medicine to cure all diseases; death being the allotted end of all human beings, will doubtless take place through the means of disease. But the cases in which medicine is useless, are yet much fewer than is supposed. Consumption is in a large number of cases a curable disease; and if the contrary appears to be the case, it is only because from its insidious approach it is usually after organs essential to life have been destroyed that relief is sought. Indeed, there are few diseases in their nature incurable; they mostly become so by neglect, and of these the number is daily becoming smaller.

When a cure is not to be effected, there still remains the alleviation of suffering, the cheering and sustaining of the restless sufferer under disease, which are among the most precious services which man can render to his suffering fellow-man.

Such are the services which the medical profession throughout the civilized world daily renders. In every climate, in every place, wherever disease is found, there is a physician strving to relieve it; and whether successful or not, his mission is nevertheless divine and charitable, soothing and consoling beyond the power of words to express. Such is the science you profess, and so noble is the work on which you are bound. Beware of them who would draw you from it, or make you undervalue its ministrations. They are disappointed candidates for eminence in some useful way, narrow minds which cannot embrace a subject so extensive, or apply in practice an art requiring so much skill.

Medical science embraces every known method of curing disease. Whatever may be the principle, if it be found useful it is adopted. The true physician, therefore, cannot select a partial class of remedies exclusively, or devote himself to a single idea to the rejection of all others. He seeks out and adopts every improvement; his motto is, progress; his course is onward, but he advances by the path of experiment and induction, not by that of imagination or conjecture.

But whence comes it that empiricism thrives, that false theories prevail, that absurd systems are built up? It results from two causes: first, the power of falsehood, which, by means of the facilities of giving notoriety and practising imposition, is enabled to fatten and thrive upon public credulity. It is useless to expect that this will ever be otherwise, so long as knavery and folly exist together in the world.

But the second cause is of a different nature, and is susceptible to a great degree of being remedied. It consists in the wide difference between medicine as a science, and its application to practice. If, as a general rule, patients when they called upon a physician, received all the relief which the art of medicine is capable of affording, there would, it is probable, be but little reason to complain of the public preference to quackery in so large a number of cases. But it happens, unfortunately, that this so far from being the case, is the rare exception. How few do we find thoroughly skilled in a single branch of the art. How much more rare to find one acquainted with all. But the public are obliged to judge of it from what they see of its application to practice. Every false diagnosis, every failure to arrive at the most appropriate remedy, every error of judgment, is by them circulated as an evidence of the uncertainty or insufficiency of medicine. Here we see that a great part of the evils of which we complain, a large proportion of the inconveniences we suffer, exist in the profession itself. For if we believe that the science of medicine has attained a high degree of perfection, and that its skilful and qualified practitioners are among the most useful of men, we must admit that the unskilful and ignorant are its opprobrium, and fully as likely to be nuisances as useful in their vocation.

These facts have long been felt, and so much so of late that, a general and combined effort, through the medium of a National Medical Association, has been made for their removal. What will be the result of its action, it is difficult to say; whether it will accomplish the work of reform, or whether it will but represent the conservative influence of the profession, remains to be seen.

The recommendation to lengthen the lecture terms—the most important it has yet made—seems to be regarded in many quarters as of doubtful propriety. It is in imitation of the courses of European schools, where the instruction, extending through a longer period of time; is composed of about the same number of lectures as with us. But in their case the attention of the student is in the mean time occupied by attendance upon hospital practice and private courses, so as to fill the whole period of time fully as much as it is occupied during the few months in this country. Whether the increase of the time of attending lectures, without any other additional facilities for obtaining instruction, is likely to prove very beneficial, is extremely doubtful. It would seem that the lectures already occupy a sufficient share of the time and attention of the student. They are aids, but by no means substitutes for study, dissections, and observations at the bed side of the patient; and it would seem that these latter should be the parts of medical education to whose improvement attention should be more especially directed. It is not theoretical, but practical knowledge and skill which is deficient. No doubt a proper acquaintance with medical literature is desirable; no doubt an attendance upon lectures greatly facilitates the acquisition of knowledge; yet when these are unaccompanied by clinical teaching, they are far from making practical physicians.

The art of observation, which lies at the foundation of all practical skill, is learned and can only be acquired at the bed side. Here, if we mistake not, lies the great evil and defect of our system of education. It is not that our lectures are not well attended, or that as a general rule the lectures are not sufficiently instructive; it consists in a want of the means of familiarizing the student with the forms of disease—in a habit of passing too rapidly and superficially over the different branches he should pursue.

The plan for a national association of delegates, seems to have been a fortunate one. Even whether the measures it adopts are sufficient to remedy the evils complained of or not, it will have the advantage of arousing public attention, and of directing it to the proper means of effecting that object. It is here, if we mistake not, that the source of improvement is to be found. If physicians are not properly educated at the present time, it is assuredly due in a great measure to the fact that public opinion in the profession has been wrong on this point, and young men have been taught to believe that two or three years' study in the office of a physician, and attending a course of lectures, was not only sufficient to enable them to "get along," but was a liberal allowance of instruction. It certainly was a great improvement on the system which preceded it, for then nothing more was required than that a student should apply to a judge of a court, who in the exercise of his discretion could grant a license to practice. At that time a large number of practitioners never witnessed dissections, and there were even many who never saw a skeleton. If we compare the period of which we are speaking—not by any means remote—when medical books in circulation were scarce and imperfect, when medical schools were few and distant from each other, and the attendance upon a course of lectures a rare exception, and hospital attendance almost unnown, with the present, in which the press teems with elaborate works, in which schools are multiplied and attendance upon two courses of lectures with frequent dissections and clinical instruction, are required, surely we cannot fail to perceive the rapid advances which have been made, nor to look forward to the future with confident and well founded anticipation of further improvement.

Those who complain of the degenerate state of the profession in recent times, must, it seems to us, have failed to imbibe its spirit. They cannot be aware of the vast strides which it has almost yearly made; it is they alone who are benighted.

Our own country has shared honorably and largely in the general activity and improvements, although its medical character and literature are far from having attained that high national standard which they should acquire. We are dependent too much on foreign works, even as text books; but there are indications of a favorable change in this respect.

The western states have been the theatre of far greater advancement in the character and intelligence of the medical profession, than any other part of this country. From having had a few years since but two or three medical schools, there are at present at least a dozen; and without instituting a comparison between these and other schools, we may safely claim that the teachers in them are as capable of teaching practical medicine in the diseases of the country in which they reside, as those at a distance who have never seen them; and they give advantages of public instruction yearly to hundreds of young men who otherwise would be entirely deprived of it. The idea of a country or a part of a country being dependent upon another for its educated men in any branch of science, implies an inferiority in intelligence which is not for a moment to be tolerated as applied to the west.

The statement has recently been made by a Dr. Holmes, Professor in a not very flourishing medical school at Boston, that the multiplication of medical schools at the west is doing great mischief in the profession. Whether this opinion was formed after a full consideration of the wants of the western states, and an acquaintance with the schools themselves, or whether it was but the expression of a prevalent impression, founded upon what the west was some years since, we have not the means of knowing; but we feel assured in either case that it cannot be sustained after considering the facts of the case. The western states have heretofore been supplied, to a very considerable extent, with their medical practitioners from the country schools of New England. These setting aside the fact that their professors possessed no acquintance with the peculiar diseases of this region, afforded no opportunity for clinical instruction, and in many cases their rules admitted of graduation after two courses of lectures, both attended during the same year, without reference to the term of study. Under these circumstances, medical schools have been established in various cities and towns of the western states, possessing every facility for affording medical instruction. The right to do so is clear, and the good policy of this course is equally evident.

For a country, possessing all the advantages for containing a large population, calculated from its extent and situation to be the centre and great body of the republic of which New England will soon be but a a small appendage, inhabited by a population composed of a fusion of the different European races, and brought, under favorable circumstances, to a degree of activity and enterprise unequalled elsewhere—for such a country, with all its advantages, to be dependent upon some villages a thousand miles off for its physicians, would certainly present an anomaly in the general order of things, which nothing but an inability to observe and teach successfully could explain. We need not say that no deficiency of this kind exists, and that the western states have abundant talent of native growth, and names among her professional men known and deservedly honored throughout the whole country.

We are not of the number who think that the increase of medical schools is likely to be attended with any very injurious effects upon the public or the profession. If it be true that the great difficulty in the way of the profession consists in a want of sufficient qualifications, it seems but natural to suppose that by the increase of schools a larger number of persons might be qualified to perform the duties with success.

It is certain that until some mode is adopted by which to open the roads to eminence equally to the profession at large, no other remedy for the evils of monopoly can be found but the indefinite multiplication of medical schools. Nor does it seem probable that the number is likely to exceed the wants of the community to so great an extent as many suppose. In the new states, the labors of the physician are extremely arduous; they are often so severe as speedily to impair his health, and the inducements to draw him from professional to other occupations are numerous. The fields for the exercise of professional skill are daily increasing in extent. But a few years since, the place we inhabit was on the extreme verge of civilization, and stretching far away to the west was a desert scarcely trodden by the foot of civilized man. Now the emigrant turns from our crowded streets, and well-peopled prairies to the far distant shores of the Pacific ocean, where his hopes, taught by the recent history of the western states, paint cities, towns, and a thriving land—the home of freedom and the arts—as about to rise and expand before his view. To you, young gentlemen, about to enter upon the practical duties of life in a profession so useful, honorable, and charitable as ours, life seems to present attractions not often found in similar circumstances. The field of usefulness is rich—vast as the ambition of man can desire. Your life, if you worthily follow your profession, is to be one of perpetual charity, of daily relief to suffering. Let me urge you never to forget those pure and honorable principles which should ever guide and characterize men entrusted with so important an office—fidelity and strict attention to those entrusted to your care, respect for your professional brethren, and above all, a regard for the character and usefulness of the profession. Your character and interests are henceforth identified with it. In proportion as you devote yourself to it, will be the return you may expect to receive. Set your mark high. Fear no obstacles ; let the one object of eminence and usefulness be always before your mind. Whatever you determine on now, if followed out with suitable perseverance, you will scarcely fail to accomplish. Whatever may be the result, no pursuit is more worthy of occupying your lives than the acquisition of knowledge and its application to the relief of human suffering.

For us who have aided you, according to our means and ability, in your progress thus far, our desire for your welfare and our efforts for the dissemination of knowledge and correct principles, will not be relaxed but followed up with renewed vigor. It is now seven years since the germ of our medical college was planted. Six individuals were found willing to listen to the teachings of a private course at that time upon a single seat; the next year another was added, and the third year some twenty persons were in attendance upon our course. By some these early efforts were regarded as premature, by others as altogether misplaced ; yet the progress of events has shown that the time and place were well chosen. Step by step has the school advanced, until its alumni constitute a large body of the most respectable practitioners of a wide extent of country. Their students constitute our classes

Page(s) missing in a great measure; and our infant institution has already acquired a developement which is a guarantee of its future advancement. It is associated with the destinies of a great and powerful city, and its prosperity and continuance will be commensurate with her growth and duration. It can never perish. Like a ship entrusted to the sea with bright sunshine and smiling skies, in its course it must meet with storms; the winds may rage against it, the waves may beat upon it, dark clouds may gather above, and rocks rise beneath it, but it will come in safety through every danger to the protected waters of the distant haven.

